# Changes in sexual experiences and sexual satisfaction during pregnancy: data from a Greek secondary hospital

**DOI:** 10.11604/pamj.2020.37.312.26813

**Published:** 2020-12-03

**Authors:** Konstantinos Zacharis, Eleni Chrysafopoulou, Stavros Kravvaritis, Theodoros Charitos, Anastasia Fouka

**Affiliations:** 1 Department of Obstetrics and Gynaecology, General Hospital of Lamia, Lamia, 35131, Greece

**Keywords:** Sexuality, sexual experiences, sexual activity, sexual satisfaction, pregnancy

## Brief

Pregnancy is a time of physical and psychological changes, and in conjunction with various social, cultural, religious, and emotional influences, it may affect a woman´s sexuality and, by extension, a couple´s sexual activity for its duration [1-2]. Doctors and midwives often need to advise pregnant women and their partners on these potential changes during pregnancy [3]. The purpose of this study is to determine Greek women´s sexual habits during pregnancy and mark any differences between those and their sexual activity when not pregnant. During the period of January-May 2018, 80 Greek women who had recently given birth filled in anonymously a self-administered written questionnaire upon exiting the Maternity Clinic of Lamia´s General Hospital. The study was approved by the hospital´s Institutional Review Board. This questionnaire included demographic data and questions on the women´s sexuality and sexual activity during pregnancy. The statistical analysis and correlation between their answers followed.

Eighty women, aged 15-47 years old (average age: 28 years) answered the questionnaire while the ages of their partners ranged from 15 to 51 years old. For 39 women (48.75%) it had been their first pregnancy, for 26 women (32.5%) their second, for 13 women (16.25%) their third, and only two of the women (2.5%) already had three or more children before that pregnancy. The general characteristics of our sample are summarized in Table 1. Out of the 80 new mothers, 63 (78.75%) said that they had had sexual contact during pregnancy. Out of these 63 women, 52 (82.54%) reported a reduced frequency of sexual contact as compared to sexual contact outside of pregnancy; 15.87% had sexual contact only during the first trimester of their pregnancy, 30.16% had sexual contact during the first and second trimesters, 53.97% reported that they had sexual contact throughout their pregnancy. It should be noted that for 11 new mothers (17.46%), 8 of whom had been pregnant to their first child, there was no change in the frequency of their sexual contact. However, the percentage of new mothers who reported no sexual contact for the duration of their pregnancy was 21.25%, 64.70% of whom said that their sexual drive had decreased, while the sexual desire of their partners had decreased in 47%.

**Table 1:** general characteristics

	Parity
**Para 1**	39/80 (48.75%)
**Para 2**	26/80 (32.5%)
**Para 3**	13/80 (16.25%)
**Para ≥4**	2/80 (2.5%)

The frequency of sexual contact reported is thus summarized: 1 or more times per day was 4.77%; approximately 3 times per week was 28.57%; <1 time per week was 26.98%; <2-3 times per month was 39.68%. Regarding the type of sexual contact, the percentages were as follows: exclusively vaginal intercourse: 49 in 63 was (77.77%); vaginal and oral intercourse: 7 in 63 (11.11%); vaginal and anal intercourse: 2 in 63 (3.18%); vaginal, anal, and oral intercourse: 2 in 63 (3.18%); other form of contact (mutual masturbation, stroking): 3 in 63 (4.76%). Moreover, 85.71% of the women who had had sexual contact during pregnancy had not used condoms. It should also be emphasized that 19.04% said that they had enjoyed sexual contact more during pregnancy, while on the other hand 52.38% said that they enjoyed it less. Finally, 28.58% of women reported no difference between sexual contact during pregnancy and sexual contact outside it.

The main findings of our study regarding sexual experiences and sexual satisfaction during pregnancy are shown in Table 2.

**Table 2:** frequencies

	Sexual contact during pregnancy	Reduction in frequency of sexual contact during pregnancy	Reduction in women´s sexual drive during pregnancy	Reduction in partner´s sexual drive	Condom use during pregnancy	Women´s sexual pleasure during pregnancy
Yes	63/80 (78.75%)	52/63 (82.54%)	11/17 (64.70%)	8/17 (47%)	9/63 (14.29%)	12/63 (19.04%)
No	17/80 (21.25%)	11/63 (17.46%)	6/17 (35.30&)	9/17 (53%)	54/63 (85.71%)	33/63 (52.38%)

The analysis of our study findings shows that Greek women continue to have sexual contact during pregnancy, although both their sexual desire and the frequency of said contact are markedly reduced. They are not afraid of having sexual contact throughout pregnancy, and they prefer vaginal intercourse. The decreased frequency of sexual contact appears to be unrelated to the number of previous pregnancies, as the answers of the women who had been pregnant with their first child and the women who had had children previously did not diverge. Pregnancy is a state which negatively affect the quality of sexual experiences between couples. A normal sex life during pregnancy is key for the partners to develop into parents. Studies have shown that there is a negative correlation between pregnancy and sexual activity [4], a fact confirmed by our study findings as well.

In addition, sexual activity during pregnancy may be influenced by physiological factors, such as aching of the lower extremities, back pain, cramps, and constipation, as well as other factors, such as woman´s age, level of education and gestational age [5]. On the other hand, one study regarding the effect of pregnancy awareness on sexual activity during the first trimester showed that the sexual activity of women who were aware that they were pregnant was significantly less frequent than that of women who were unaware of their pregnancy [6]. Prior to the pregnancy, couples are not informed on the ways in which they could manage their sexual life and cover their sexual needs during pregnancy. At the same time, doctors and midwives did not systematically assess pregnant women´s and their partners´ sexual health [7].

Therefore, sexual education programs pose a challenge to clinicians with conservative views on sexuality. The individualized and in person sexual education of couples is beneficial to their sexual well-being during pregnancy and postpartum. It has been proven that such educational programs improve the sexual life of the couples who participate in them, both in terms of frequency and of satisfaction [7]. Based on the aforementioned, we conclude that doctors and other professionals of obstetrics (midwives, doulas) ought to inform couples on the psychological and sexual changes which may occur during pregnancy [8-10]. As regards to the couples themselves, it is important that they understand the normal fluctuations of sexual interest and gradual decrease of their sexual desire. Individual counselling should be provided by personnel with the appropriate expertise in order to both safeguard the pregnancy and minimize any stress regarding the couple´s sex life [11-13].
